# Sex- and Subtype-Specific Analysis of *H2AFX* Polymorphisms in Non-Hodgkin Lymphoma

**DOI:** 10.1371/journal.pone.0074619

**Published:** 2013-09-17

**Authors:** Karla L. Bretherick, Johanna M. Schuetz, Lindsay M. Morton, Mark P. Purdue, Lucia Conde, Richard P. Gallagher, Joseph M. Connors, Randy D. Gascoyne, Brian R. Berry, Bruce Armstrong, Anne Kricker, Claire M. Vajdic, Andrew Grulich, Henrik Hjalgrim, Karin E. Smedby, Christine F. Skibola, Nathaniel Rothman, John J. Spinelli, Angela R. Brooks-Wilson

**Affiliations:** 1 Canada’s Michael Smith Genome Sciences Centre, BC Cancer Agency, Vancouver, British Columbia, Canada; 2 Department of Medical Genetics, University of British Columbia, Vancouver, British Columbia, Canada; 3 Division of Cancer Epidemiology and Genetics, National Cancer Institute, Bethesda, Maryland, United States of America; 4 Division of Environmental Health Sciences, School of Public Health, University of California, Berkeley, California, United States of America; 5 Cancer Control Research, BC Cancer Agency, Vancouver, British Columbia, Canada; 6 Division of Medical Oncology and Centre for Lymphoid Cancer, BC Cancer Agency, Vancouver, British Columbia, Canada; 7 Department of Pathology and Centre for Lymphoid Cancer, BC Cancer Agency, Vancouver, British Columbia, Canada; 8 Department of Pathology, Royal Jubilee Hospital, Victoria, British Columbia, Canada; 9 Sydney School of Public Health, The University of Sydney, Sydney, Australia; 10 Adult Cancer Program, Lowy Cancer Research Centre, Prince of Wales Clinical School, Faculty of Medicine at the University of New South Wales, Sydney, Australia; 11 The Kirby Institute for infection and immunity in society, University of New South Wales, New South Wales, Australia; 12 Department of Epidemiology Research, Statens Serum Institut, Copenhagen, Denmark; 13 Unit of Clinical Epidemiology, Department of Medicine, Solna, Karolinska Institute, Stockholm, Sweden; 14 School of Population and Public Health, University of British Columbia, Vancouver, British Columbia, Canada; 15 Department of Biomedical Physiology and Kinesiology, Simon Fraser University, Burnaby, British Columbia, Canada; Faculty of Medicine, University of Porto, Portugal

## Abstract

*H2AFX* encodes a histone variant involved in signaling sites of DNA damage and recruiting repair factors. Genetic variants in *H2AFX* may influence risk of non-Hodgkin lymphoma (NHL), a heterogeneous group of lymphoid tumors that are characterized by chromosomal translocations. We previously reported that rs2509049, a common variant in the promoter of *H2AFX*, was associated with risk for NHL in the British Columbia population. Here we report results for 13 single nucleotide polymorphisms (SNPs) in 100 Kb surrounding *H2AFX* in an expanded collection of 568 NHL cases and 547 controls. After correction for multiple testing, significant associations were present for mantle cell lymphoma (*p*=0.007 for rs604714) and all B-cell lymphomas (*p*=0.046 for rs2509049). Strong linkage disequilibrium in the 5 Kb upstream of *H2AFX* limited the ability to determine which specific SNP (rs2509049, rs7759, rs8551, rs643788, rs604714, or rs603826), if any, was responsible. There was a significant interaction between sex and rs2509049 in the all B-cell lymphomas group (*p*=0.002); a sex-stratified analysis revealed that the association was confined to females (*p*=0.001). Neither the overall nor the female-specific association with rs2509049 was replicated in any of four independent NHL sample sets. Meta-analysis of all five study populations (3,882 B-cell NHL cases and 3,718 controls) supported a weak association with B-cell lymphoma (OR=0.92, 95% CI=0.86-0.99, p=0.034), although this association was not significant after exclusion of the British Columbia data. Further research into the potential sex-specificity of the *H2AFX*-NHL association may identify a subset of NHL cases that are influenced by genotype at this locus.

## Introduction

Non-Hodgkin lymphomas (NHL) are a histologically diverse group of neoplasms of lymphoid origin that vary in severity of clinical behavior from indolent to very aggressive. NHLs can be broadly divided into tumors of B-cell or T-cell origin, and each of these can be further classified based on clinical features, pathology, histology and/or genetic indicators into one of over 40 subtypes [1].

A key characteristic of B-cell development is the creation of a diverse repertoire of immunoglobulin receptors able to mount an immune response to a vast assortment of foreign antigens [2]. This diversity is accomplished by three processes that alter immunoglobulin genes: V(D)J recombination, somatic hypermutation and class switch recombination (reviewed in 2). All of these processes require maintenance of DNA integrity and in particular, both *V(D*)* J* and class switch recombination require repair of double stranded DNA breaks [3]. Aberrant resolution of these breaks might lead to oncogenic chromosomal translocations by juxtaposition of genes that confer a growth stimulating or anti-apoptotic effect with DNA elements that lead to high or inappropriately timed expression in lymphoid cells [2]. It is therefore not surprising that reciprocal chromosomal translocations involving the immunoglobulin loci are a characteristic of many NHLs [2]. The tendency for some NHL subtypes to have translocations may imply an underlying defect in the cellular systems that protect against them, such as genes involved in DNA repair or surveillance for damaged DNA. Attenuation of the process of double-stranded break repair due to genetic variation in the genes involved may lead to increased translocations and influence NHL risk.

H2AX is a non-canonical histone which replaces 2-25% of histone H2A molecules that compact DNA into the nucleosome, the basic unit of chromatin organization (reviewed in 3). H2AX is involved in signaling the presence of double stranded breaks, recruiting DNA repair factors and preventing DNA breaks from progressing to translocations. In a process primarily mediated by activated ATM, double stranded breaks prompt phosphorylation of a highly conserved C-terminal serine residue that is unique to the H2AX histone [4]. Phosphorylated H2AX (γH2AX) recruits DNA damage repair factors to the sites of double stranded breaks and initiates a signal cascade that amplifies and expands the DNA repair signal (reviewed in 3). H2AX phosphorylation is such an integral part of the double strand break repair process that staining for γH2AX foci is a frequently used indicator for visualizing the sites of DNA damage within a cell. H2afx deficient mice have reduced DNA repair efficiency and show elevated levels of chromosome instability, DNA repair defects and tumorigenesis [5-7]. Specific to B-cell development, H2AX is required for efficient resolution of the double stranded breaks induced during class switch recombination [8] and stabilization of DNA strands to prevent progression to chromosome breaks during V(D)J recombination [9]. Significant roles for H2AX in both *V(D*)* J* and class switch recombination suggest that optimal H2AX function may be particularly important in preventing tumor formation in lymphoid cells.

We previously reported that a single nucleotide polymorphism (SNP), rs2509049, in the promoter region of the H2AX gene, *H2AFX*, was associated with NHL [10]. Specifically, rs2509049 was associated with translocation-prone follicular (FL) and mantle cell (MCL) lymphomas, but not with diffuse large B cell lymphoma (DLBCL), consistent with a role for H2AX in prevention of translocations. Subsequently, other groups have reported that *H2AFX* genetic variants are associated with breast cancer [11], glioma [12] and DLBCL [13] but not with bladder cancer [14]. However it remains unknown which variant or group of variants at the *H2AFX* locus contributes to the risk of malignancy.

To explore, confirm and characterize the *H2AFX* association with NHL, we analyzed genotypes of 13 SNPs in a 100 Kb region surrounding *H2AFX* in constitutional DNA of an expanded collection of 568 NHL cases and 547 control individuals from the British Columbia (BC) population and in four independent NHL sample sets: 1) the Scandinavian Lymphoma Etiology study (SCALE), 2) a population-based case-control study in San Francisco (SF), 3) NHL patients and controls collected as part of the National Cancer Institute – Surveillance, Epidemiology and End Results study (NCI-SEER) in the United States and 4) a population-based case-control study of NHL patients in New South Wales and the Australian Capital Territory, Australia (NSW).

## Materials and Methods

### Ethics Statement

This study was approved by the joint University of British Columbia/British Columbia Cancer Agency Research Ethics Board and written informed consent was obtained from all participants.

### Study population

The study population has been previously described [15,16], and the case and control samples genotyped in this study have been described in detail [17]. Briefly, DNA was obtained from 797 cases (20-79 years of age) and 790 controls frequency matched for age, sex and region (Vancouver or Victoria), collected as part of a population based study of NHL in British Columbia from March 2000 to February 2004.

### Genotyping

Initially, 16 SNPs in a 100 Kb region encompassing the *H2AFX* region were selected for genotyping (Table S1). These included 8 tagSNPs [18] chosen to represent the variation in SNPs genotyped by HapMap in the CEU population, 3 SNPs chosen from literature reports of associations with *H2AFX* [10,11] and an additional 5 SNPs added to further saturate the regions 5 Kb upstream and downstream of *H2AFX*. One SNP failed Illumina genotyping design. The remaining 15 SNPs were genotyped at The Centre for Applied Genomics, at the Hospital for Sick Children in Toronto, Canada as part of a larger Golden Gate assay (Illumina, San Diego, CA) which has been described previously [17]. Genotypes were assessed using Genome Studio version 2009.1 (Illumina, San Diego, CA).

Prior to analysis, all SNPs and samples included in the assay were subject to extensive quality control previously described in detail [17]. SNP quality control included exclusions based on: GenCall score (< 0.25); GenTrain score (<0.4); poor or abnormal genotype clustering; discrepancies between 53 pairs of duplicate samples; poor call rates (<95%); and deviation from Hardy Weinberg equilibrium (HWE; p<0.001) in European-ancestry controls. Although all 15 *H2AFX* SNPs met overall quality control requirements, 2 SNPs (rs28990980 and rs603826) were subsequently excluded from analysis (Table S1). rs28990980 was excluded due to a very low minor allele frequency (0.002) in control samples; and rs603826, although passing multiple testing-corrected HWE cutoffs at the overall quality control stage, had an uncorrected HWE p value suggesting a departure from HWE (p=0.007). Examination of the sequence surrounding this variant revealed the presence of SNP rs10892330 within 3 base pairs of rs603826, which had not been recognized at the time of assay design. As this nearby SNP may interfere with Illumina probe binding and may be responsible for the observed deviation from HWE, rs603826 was excluded from analysis. Thus, after genotyping and quality control, 13 SNPs remained for analysis.

Sample quality control has been described previously [17]. It included: exclusions based on call rate (<0.98); exclusions based on discrepancies in sex and race between what was reported for a sample and what was supported by sample genotypes; and exclusions based on unexpected relatedness between samples revealed by SNP analysis [17]. These quality control measures resulted in exclusion of 176 samples leaving 1411/1587 samples remaining. One additional case was excluded from analyses due to diagnoses of both B-cell and T-cell lymphomas. All analyses reported here were restricted to 568 NHL cases and 547 controls (1115 samples) who reported that all four grandparents were of European-descent and for whom genotype data supported European ancestry [17].

### Replication study populations and genotyping

The four study populations used to replicate findings have been described previously. All genotyping platforms used are highly accurate and cases and controls within each study were genotyped in an identical manner.

The Scandinavian lymphoma etiology study (SCALE) is a population-based case-control study of individuals (18-75 years old) collected in Denmark and Sweden between 1999 and 2002 [19]. rs2509049 genotypes were available for 4294 samples genotyped using Sequenom technology and SpectroTYPER RT3.4 software (Sequenom Inc., San Diego, CA) as described [20]. Samples (N=46) who did not report that both parents were born in Europe were excluded, leaving 1871 controls and 2376 NHL cases (2183 of B-cell origin) for analysis.

The San Francisco study (SF) is a population-based case-control study of individuals (20-84 years old) collected in the San Francisco Bay area between 2001 and 2005 [21]. Genotypes for rs2509049 were imputed using the BEAGLE 3.0.3 software [22] based on haplotype information from unrelated HapMap-II CEU samples. SNPs imputed with maximum posterior probability < 0.9 were set to missing and those with >10% missing rate were further excluded. The analyses reported here were limited to 737 controls and 664 cases who reported non-Hispanic white race and for whom genotype data supported non-Hispanic white race [21].

The National Cancer Institute – Surveillance, Epidemiology and End Results (NCI-SEER) study is a case- control study of NHL cases (20-74 years old) identified in Detroit, Iowa, Los Angeles, or Seattle SEER registries between 1998 and 2000 and population controls identified by random digit dialing random digit dialing (<65 years) and from Medicare eligibility files (>65 years) [23]. rs2509049 genotypes determined by Fluidigm technology (Fluidigm Corporation, San Francisco, CA) were available for 455 controls and 516 NHL cases. The analyses reported here were confined to 378 controls and 442 NHL cases (373 of B-cell origin) who self-reported non-Hispanic white race.

The New South Wales (NSW) study is a population-based case-control study of NHL cases (20-74 years old) identified in NSW or the Australian Capital Territory (ACT) between 2000 and 2001 and matched controls randomly selected from the NSW and ACT electoral rolls [24]. rs2509049 genotypes determined by Fluidigm technology (Fluidigm Corporation, San Francisco, CA) were available for 268 controls and 245 NHL cases. Analyses reported here were confined to 264 controls and 239 NHL cases (218 of B cell origin) who self-reported non-Hispanic white race.

### Statistical analysis

BC cases of each NHL subtype were compared separately to all BC controls. Odds ratios (OR) and corresponding 95% confidence intervals (CI) were estimated by logistic regression performed with SVS Suite 7 (Golden Helix, Bozeman, MT). *P*-values for an additive model were calculated for a full model including the SNP of interest vs. a reduced model which accounted for age group (in 5 year increments), sex and region of residence; uncorrected p values are indicated in tables as *p*. Full scan permutations carried out in SVS (10,000 permutations) were performed to account for multiple testing; corrected p values are indicated as *p adj*.

Since the association of *H2AFX* variants with glioma is reportedly stronger in males [12], we hypothesized that effect of *H2AFX* genotype on NHL risk may also be influenced by sex. To assess interaction with sex, the SNP with the most significant p value within each NHL subtype was chosen to represent the gene for that subtype [17] and was analyzed by logistic regression comparing a full model including sex*SNP as an interaction term, to a reduced model with sex, age group, region of residence and SNP. For subtypes in which this analysis was significant, the data was stratified by sex and logistic regression separately in the female and male strata (correcting for age group and region of residence) for all 13 SNPs.

Linkage disequilibrium in the cases and controls was determined using Haploview v4.2. Haplotype blocks were predicted with Haploview 4.2 using 95% confidence bounds on D’ [25] with the following parameters: CI minima for strong linkage disequilibrium (LD) of 0.7-0.98, upper CI for strong recombination of 0.90, fraction of strong LD in informative comparisons of at least 95% and exclusion of SNPs with a minor allele frequency < 0.10. Haplotype frequencies in cases and controls were determined with SVS Suite 7 using the expectation-maximization method and logistic regression was performed for haplotypes with frequencies >0.01, as described for individual SNPs.

Analyses of independent study populations for replication were performed in R version 2.15.1 [26] on individuals of European ancestry or white race for those subtypes or groups for which at least two studies had genotypes for more than 100 samples: the DLBCL and FL subtypes and a group encompassing all B cell lymphomas. Study-specific ORs and 95% CIs were estimated by logistic regression, with P values for an additive model determined by comparing the full model to a reduced model which included study-specific variables described in Table S2. Heterogeneity between ORs from different studies was assessed using Cochran’s Q test performed in with rmeta version 2.16 [27]. ORs without significant heterogeneity between studies (Q>0.10) were combined by meta-analysis under a fixed effects model. For analyses with significant heterogeneity in ORs between studies, a random effects model was used.

## Results

The characteristics of the 568 NHL cases and 547 controls from the BC study population who met quality control criteria and were included in analyses are described in Table 1. Although controls were frequency-matched to cases by age, sex and region in the study overall, cases of European descent were more likely to be male, older and resident of Vancouver than controls of European descent.

**Table 1:** Characteristics of the BC study population.

	**Controls**		**Cases**		
	**N**	**(%**)	**N**	**(%**)	***p***
**Sex**					*0.032*
Male	279	(51)	327	(58)	
Female	268	(49)	241	(42)	
**Age (years**)					*0.049*
20-49	119	(22)	87	(15)	
50-59	122	(22)	137	(24)	
60-69	152	(28)	165	(29)	
70+	154	(28)	179	(32)	
**Region**					*0.01*
Vancouver	392	(72)	446	(79)	
Victoria	155	(28)	122	(21)	
**Subtype**					
All B-cell lymphomas			523	(92)	
DLBCL			148	(26)	
FL			165	(29)	
MZL/MALT			55	(10)	
MCL			40	(7)	
SLL/CLL			35	(6)	
LPL			34	(6)	
Misc. B cell			46	(8)	
T-cell and NK-cell lymphomas			45	(8)	
**Total**	547		568		

Abbreviations: DLBCL, diffuse large B-cell lymphoma; FL, follicular lymphoma; MZL/MALT, marginal zone lymphoma/mucosa-associated lymphoid tissue lymphoma; MCL, mantle cell lymphoma; SLL/CLL small lymphocytic lymphoma/chronic lymphocytic leukemia; LPL, lymphoplasmacytic lymphoma

### Subtype-specific analysis

**Figure 1:**
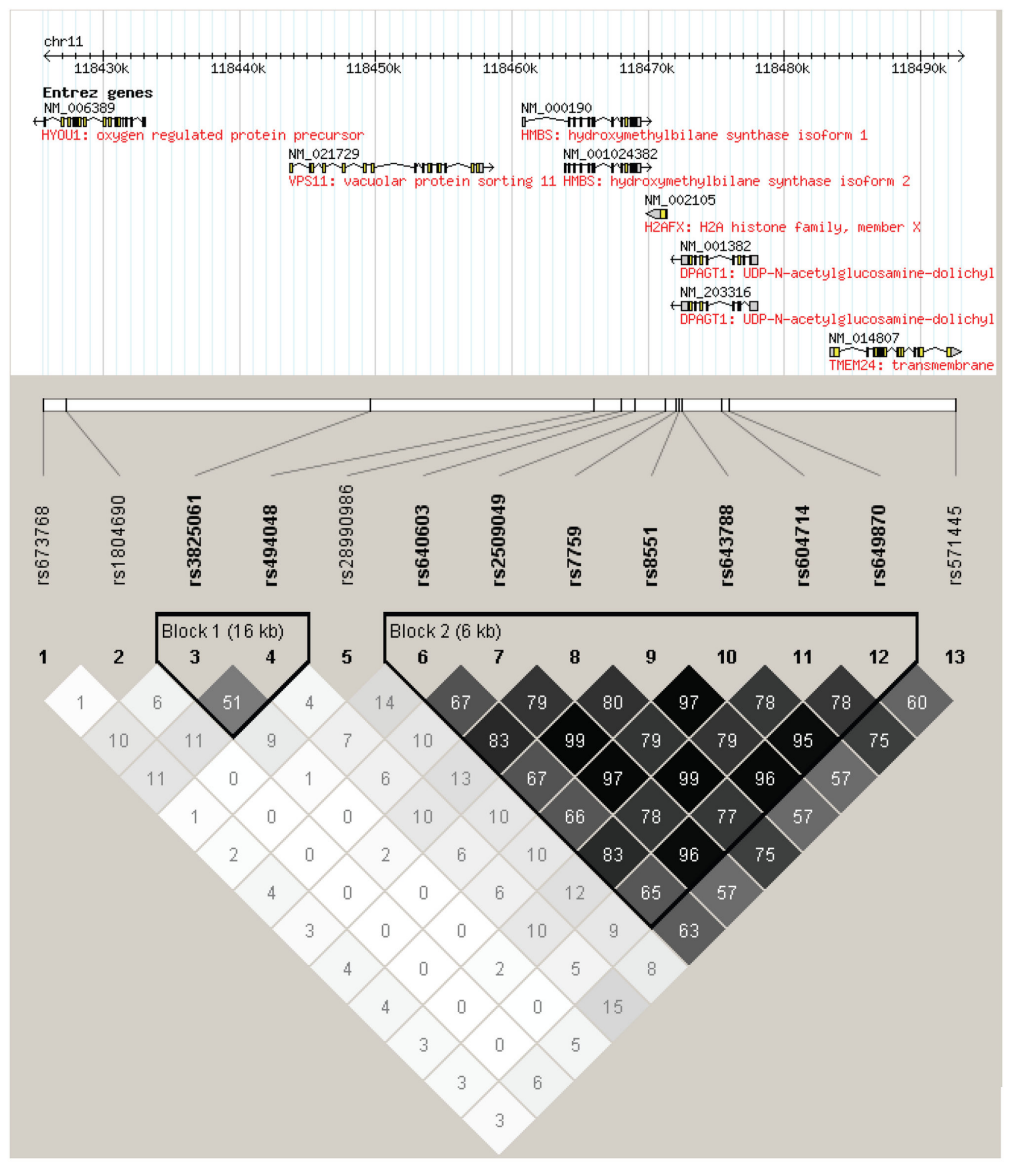
Linkage Disequilibrium for 13 SNPs in 1115 cases and controls in the BC population. Chromosomal coordinates (Hg18) and Entrez genes are mapped relative to the 13 SNPs analyzed in this study. The linkage disequilibrium plot shows *r* ^2^ values; predicted haplotype blocks are outlined in black. Figure created with Haploview version 4.2.

Subtype-specific association results for 13 SNPs within 100 Kb of *H2AFX* are summarized in Tables 2 and 3. rs2509049 was associated with the FL and MCL subtypes and with the all B-cell group; however, only the associations with MCL and the all B-cell group remained significant after correction for multiple testing. Additional SNPs in linkage disequilibrium (LD) with rs2509049 (Figure 1) were also associated with the FL and MCL subtypes and the all B-cell group, with the most significant *p* values observed for the association of rs604714 with MCL. rs1804690, a SNP located more than 40 Kb downstream of *H2AFX* and not in LD with rs2509049, was associated with NHL in the DLBCL subtype and the all B-cell group, and remained significant after multiple testing correction in the all B-cell group. There were no associations with any of the SNPs for the MZL/MALT or T/NK cell lymphoma subtypes.

**Table 2:** Subtype-specific association results for 13 SNPs in the BC population.

		**Controls (N=547**)		**DLBCL (N=148**)						**FL (N=165**)						**MZL/MALT (N=55**)					
**Variant**	**Genotype**	N*	**(%**)	N*	**(%**)	**OR**	**(95% CI**)	***p***	***padj***	N*	**(%**)	**OR**	**(95% CI**)	***p***	***padj***	N*	**(%**)	**OR**	**(95% CI**)	***p***	***padj***
rs673768	GG	397	(73)	106	(72)			*0.977*	*1.000*	118	(72)	1		*0.619*	*0.999*	40	(73)			*0.776*	*1.000*
	AG	140	(26)	41	(28)					40	(24)	0.93	(0.62-1.41)			15	(27)				
	AA	8	(1)	0	(0)					6	(4)	2.27	(0.77-6.72)			0	(0)				
rs1804690	GG	428	(78)	130	(88)	1		***0.009***	*0.066*	141	(85)	1		*0.062*	*0.361*	45	(82)			*0.546*	*0.994*
	AG	113	(21)	17	(11)	0.49	(0.28-0.85)			23	(14)	0.66	(0.40-1.07)			10	(18)				
	AA	6	(1)	1	(1)	0.58	(0.07-4.97)			1	(1)	0.46	(0.05-3.88)			0	(0)				
rs3825061	GG	226	(41)	54	(36)	1		*0.161*	*0.672*	55	(33)	1		*0.207*	*0.782*	22	(40)	1		*0.636*	*0.999*
	AG	239	(44)	64	(43)	1.13	(0.75-1.70)			88	(53)	1.55	(1.06-2.29)			24	(44)	1.08	(0.58-2.00)		
	AA	81	(15)	30	(20)	1.48	(0.88-2.48)			22	(13)	1.18	(0.67-2.06)			9	(16)	1.23	(0.53-2.85)		
rs494048	GG	119	(25)	37	(28)	1		*0.400*	*0.961*	40	(30)	1		*0.165*	*0.698*	13	(29)	1		*0.334*	*0.928*
	AG	239	(51)	69	(52)	0.98	(0.62-1.56)			68	(51)	0.85	(0.54-1.35)			24	(53)	0.94	(0.45-1.94)		
	AA	110	(24)	26	(20)	0.77	(0.44-1.37)			25	(19)	0.67	(0.38-1.18)			8	(18)	0.62	(0.24-1.59)		
rs28990986	GG	469	(86)	120	(81)			*0.355*	*0.939*	140	(85)			*0.997*	*1.000*	45	(83)			*0.582*	*0.997*
	AG	71	(13)	28	(19)					25	(15)					9	(17)				
	AA	6	(1)	0	(0)					0	(0)					0	(0)				
rs640603	GG	185	(40)	60	(45)	1		*0.494*	*0.989*	62	(47)	1		*0.070*	*0.399*	20	(44)	1		*0.254*	*0.853*
	AG	204	(44)	51	(39)	0.81	(0.52-1.24)			55	(41)	0.78	(0.51-1.19)			21	(47)	0.94	(0.49-1.83)		
	AA	79	(17)	21	(16)	0.88	(0.50-1.56)			16	(12)	0.59	(0.32-1.09)			4	(9)	0.47	(0.15-1.46)		
rs2509049	GG	141	(30)	46	(35)	1		*0.512*	*0.992*	55	(41)	1		***0.018***	*0.128*	16	(36)	1		*0.174*	*0.720*
	AG	216	(46)	58	(44)	0.87	(0.56-1.37)			53	(40)	0.62	(0.40-0.96)			23	(51)	0.92	(0.46-1.85)		
	AA	111	(24)	28	(21)	0.84	(0.49-1.45)			25	(19)	0.55	(0.32-0.95)			6	(13)	0.48	(0.18-1.30)		
rs7759	AA	169	(36)	53	(40)	1		*0.680*	*1.000*	62	(47)	1		***0.012***	*0.093*	17	(38)	1		*0.289*	*0.897*
	GA	210	(45)	55	(42)	0.85	(0.55-1.31)			54	(41)	0.68	(0.45-1.05)			24	(53)	1.1	(0.56-2.15)		
	GG	89	(19)	24	(18)	0.93	(0.53-1.62)			17	(13)	0.50	(0.27-0.92)			4	(9)	0.45	(0.14-1.40)		
rs8551	GG	170	(31)	51	(34)	1		*0.606*	*0.999*	66	(40)	1		***0.022***	*0.149*	18	(33)	1		*0.651*	*1.000*
	AG	249	(46)	65	(44)	0.89	(0.58-1.35)			70	(42)	0.72	(0.49-1.07)			26	(47)	0.97	(0.51-1.84)		
	AA	126	(23)	32	(22)	0.89	(0.54-1.47)			29	(18)	0.58	(0.35-0.95)			11	(20)	0.82	(0.37-1.82)		
rs643788	AA	165	(30)	50	(34)	1		*0.576*	*0.998*	65	(39)	1		***0.017***	*0.121*	18	(33)	1		*0.571*	*0.997*
	GA	256	(47)	66	(45)	0.86	(0.57-1.32)			71	(43)	0.70	(0.47-1.03)			26	(47)	0.92	(0.48-1.76)		
	GG	126	(23)	32	(22)	0.88	(0.53-1.46)			29	(18)	0.56	(0.34-0.93)			11	(20)	0.79	(0.35-1.76)		
rs604714	CC	203	(37)	60	(41)	1		*0.706*	*1.000*	73	(44)	1		***0.037***	*0.237*	19	(35)	1		*0.746*	*1.000*
	AC	244	(45)	61	(41)	0.85	(0.57-1.28)			71	(43)	0.81	(0.55-1.18)			29	(53)	1.24	(0.67-2.31)		
	AA	100	(18)	27	(18)	0.95	(0.57-1.60)			21	(13)	0.57	(0.33-0.98)			7	(13)	0.74	(0.30-1.85)		
rs649870	AA	164	(30)	50	(34)	1		*0.591*	*0.998*	63	(38)	1		***0.022***	*0.149*	18	(33)	1		*0.540*	*0.994*
	GA	254	(46)	65	(44)	0.85	(0.56-1.29)			73	(44)	0.75	(0.51-1.11)			26	(47)	0.94	(0.49-1.79)		
	GG	129	(24)	33	(22)	0.89	(0.54-1.47)			29	(18)	0.57	(0.34-0.94)			11	(20)	0.77	(0.35-1.72)		
rs571445	AA	162	(30)	46	(31)	1		*0.720*	*1.000*	58	(35)	1		*0.061*	*0.357*	16	(29)	1		*0.674*	*1.000*
	GA	259	(47)	71	(48)	0.97	(0.64-1.48)			79	(48)	0.86	(0.58-1.27)			29	(53)	1.15	(0.60-2.21)		
	GG	126	(23)	31	(21)	0.91	(0.54-1.52)			28	(17)	0.61	(0.36-1.01)			10	(18)	0.80	(0.35-1.86)		

* The sum of the genotypes is in some cases lower than the total number of samples for a subtype, because some samples failed Illumina genotyping for some markers.

Abbreviations: DLBCL, diffuse large B-cell lymphoma; FL, follicular lymphoma; MZL/MALT, marginal zone lymphoma/mucosa-associated lymphoid tissue; OR, odds ratio; CI, confidence interval

**Table 3:** Subtype-specific association results for 13 SNPs in the BC population.

		**Controls (N=547**)		**MCL (N=40**)						**All B-cell (N=523**)						**T-cell & NK-cell (N=45**)					
**Variant**	**Genotype**	N*	**(%**)	N*	**(%**)	**OR**	**(95% CI**)	***p***	***padj***	N*	**(%**)	**OR**	**(95% CI**)	***p***	***padj***	N*	**(%**)	**OR**	**(95% CI**)	***p***	***padj***
rs673768	GG	397	(73)	28	(70)	1		*0.467*	*0.988*	376	(72)	1		*0.638*	*1.000*	33	(73)	1		*0.858*	*1.000*
	AG	140	(26)	11	(28)	1.25	(0.60-2.61)			134	(26)	1.03	(0.78-1.36)			11	(24)	0.90	(0.44-1.84)		
	AA	8	(1)	1	(3)	1.77	(0.20-15.55)			11	(2)	1.33	(0.52-3.38)			1	(2)	1.15	(0.14-9.57)		
rs1804690	GG	428	(78)	35	(88)			*0.152*	*0.677*	450	(86)	1		***0.001***	***0.006***	36	(80)			*0.729*	*1.000*
	AG	113	(21)	5	(13)					71	(14)	0.60	(0.43-0.83)			9	(20)				
	AA	6	(1)	0	(0)					2	(0)	0.30	(0.06-1.53)			0	(0)				
rs3825061	GG	226	(41)	14	(35)	1		*0.590*	*0.998*	187	(36)	1		*0.053*	*0.315*	16	(36)	1		*0.472*	*0.986*
	AG	239	(44)	19	(48)	1.23	(0.60-2.54)			246	(47)	1.27	(0.98-1.66)			22	(49)	1.37	(0.70-2.70)		
	AA	81	(15)	7	(18)	1.24	(0.48-3.24)			90	(17)	1.35	(0.94-1.94)			7	(16)	1.27	(0.50-3.24)		
rs494048	GG	119	(25)	13	(35)	1		*0.258*	*0.865*	132	(30)	1		***0.041***	*0.250*	8	(21)	1		*0.960*	*1.000*
	AG	239	(51)	17	(46)	0.67	(0.31-1.44)			229	(52)	0.87	(0.64-1.18)			23	(61)	1.46	(0.63-3.40)		
	AA	110	(24)	7	(19)	0.59	(0.23-1.56)			82	(19)	0.67	(0.46-0.98)			7	(18)	0.95	(0.33-2.74)		
rs28990986	GG	469	(86)	39	(98)			***0.021***	*0.156*	442	(85)	1		*0.698*	*1.000*	39	(87)			*0.684*	*1.000*
	AG	71	(13)	1	(3)					78	(15)	1.20	(0.85-1.71)			6	(13)				
	AA	6	(1)	0	(0)					2	(0)	0.36	(0.07-1.83)			0	(0)				
rs640603	GG	185	(40)	24	(65)	1		***0.003***	***0.019***	208	(47)	1		***0.012***	*0.086*	19	(50)	1		*0.136*	*0.644*
	AG	204	(44)	11	(30)	0.43	(0.20-0.90)			181	(41)	0.78	(0.59-1.04)			15	(39)	0.69	(0.34-1.42)		
	AA	79	(17)	2	(5)	0.21	(0.05-0.91)			54	(12)	0.62	(0.42-0.93)			4	(11)	0.48	(0.15-1.46)		
rs2509049	GG	141	(30)	19	(51)	1		***0.003***	***0.025***	167	(38)	1		***0.006***	***0.046***	15	(39)	1		*0.424*	*0.973*
	AG	216	(46)	15	(41)	0.52	(0.25-1.07)			198	(45)	0.77	(0.57-1.04)			14	(37)	0.60	(0.28-1.29)		
	AA	111	(24)	3	(8)	0.21	(0.06-0.72)			79	(18)	0.60	(0.42-0.87)			9	(24)	0.74	(0.31-1.79)		
rs7759	AA	169	(36)	23	(62)	1		***0.001***	***0.010***	194	(44)	1		***0.007***	*0.054*	16	(42)	1		*0.556*	*0.996*
	GA	210	(45)	12	(32)	0.43	(0.20-0.89)			189	(43)	0.77	(0.58-1.03)			15	(39)	0.73	(0.35-1.54)		
	GG	89	(19)	2	(5)	0.17	(0.04-0.76)			61	(14)	0.61	(0.41-0.90)			7	(18)	0.81	(0.32-2.08)		
rs8551	GG	170	(31)	20	(50)	1		***0.004***	***0.028***	193	(37)	1		***0.016***	*0.111*	19	(42)	1		*0.296*	*0.904*
	AG	249	(46)	17	(43)	0.58	(0.29-1.14)			232	(45)	0.82	(0.62-1.08)			16	(36)	0.57	(0.29-1.15)		
	AA	126	(23)	3	(8)	0.20	(0.06-0.70)			96	(18)	0.66	(0.47-0.93)			10	(22)	0.71	(0.32-1.59)		
rs643788	AA	165	(30)	20	(50)	1		***0.011***	*0.076*	192	(37)	1		***0.014***	*0.099*	19	(42)	1		*0.247*	*0.855*
	GA	256	(47)	15	(38)	0.48	(0.24-0.97)			231	(44)	0.77	(0.58-1.01)			16	(36)	0.53	(0.27-1.07)		
	GG	126	(23)	5	(13)	0.33	(0.12-0.91)			99	(19)	0.67	(0.47-0.93)			10	(22)	0.68	(0.30-1.54)		
rs604714	CC	203	(37)	25	(63)	1		***0.001***	***0.007***	223	(43)	1		***0.025***	*0.168*	21	(47)	1		*0.262*	*0.877*
	AC	244	(45)	13	(33)	0.43	(0.21-0.87)			224	(43)	0.82	(0.63-1.07)			17	(38)	0.67	(0.34-1.30)		
	AA	100	(18)	2	(5)	0.17	(0.04-0.73)			75	(14)	0.68	(0.47-0.97)			7	(16)	0.67	(0.27-1.65)		
rs649870	AA	164	(30)	20	(50)	1		***0.002***	***0.016***	188	(36)	1		***0.015***	*0.107*	19	(42)	1		*0.228*	*0.825*
	GA	254	(46)	17	(43)	0.54	(0.27-1.07)			236	(45)	0.81	(0.61-1.07)			16	(36)	0.54	(0.27-1.09)		
	GG	129	(24)	3	(8)	0.19	(0.06-0.67)			99	(19)	0.66	(0.47-0.93)			10	(22)	0.66	(0.30-1.49)		
rs571445	AA	162	(30)	21	(53)	1		***0.001***	***0.007***	182	(35)	1		***0.023***	*0.158*	16	(36)	1		*0.209*	*0.787*
	GA	259	(47)	17	(43)	0.52	(0.27-1.03)			244	(47)	0.83	(0.63-1.09)			22	(49)	0.85	(0.43-1.68)		
	GG	126	(23)	2	(5)	0.13	(0.03-0.58)			96	(18)	0.68	(0.48-0.95)			7	(16)	0.55	(0.22-1.38)		

* The sum of the genotypes is in some cases lower than the total number of samples for a subtype, because some samples failed Illumina genotyping for some markers.

Abbreviations: MCL, mantle cell lymphoma; T/NK cell, T-cell and NK-cell lymphoma; OR, odds ratio; CI, confidence interval

This BC dataset included 214 new cases and 164 new controls in addition to 354 cases and 383 controls for which results had been reported previously [10]. From the previously reported data, only samples for which genotypes were confirmed by Illumina, Golden Gate assay were included these analyses, this excluded 33 cases and 37 controls from our original report [10] for which there was insufficient sample remaining. Analysis confined to the new B-cell lymphoma cases (N=196) and controls (N=164) was significant only for rs1804690 (OR=0.39, 95% CI=0.22-0.69, *p* =0.0007) and not for rs2509049 (OR=0.93, 95% CI=0.67-1.29, *p*=0.662). Further subtype analyses confined to the new data were not warranted due to small sample sizes.

### Sex-specific analysis

The SNPs with most significant *p* values in the DLBCL, FL and MCL subtypes were assessed for interaction with sex in that subtype. For the all B-cell group, four SNPs were assessed for interaction with sex: rs2509049, as it had the lowest *p* value in B-cell lymphomas, and rs1804690, rs7759 and rs604714 as they were being assessed in the subtype analyses (Table S3). No interactions were significant in the subtype analyses; however for the all B-cell group, rs2509049, rs7759 and rs604714 had significant interactions with sex (*p*=0.002, *p*=0.003 and *p*=0.015, respectively). The lowest ORs and most significant *p* values were observed for the rs2509049-sex interaction in the all B-cell group (OR_interaction=0.56, 95% CI=0.59-0.81, *p*=0.002). Cases and controls were therefore stratified by sex and assessed for association separately in males and females in the all B-cell group (Table 4). In males, rs1804690 was the only SNP significantly associated with B cell NHL, whereas in the females, 10 SNPs in the *H2AFX* region were associated with NHL with the most significant association being with rs2509049.

**Table 4:** Sex-specific association results for 13 SNPs in all B-cell lymphomas in the BC population.

		**Males**								**Females**							
		**Controls**		**Cases**						**Controls**		**Cases**					
**Variant**	**Genotype**	N*	**(%**)	N*	**(%**)	**OR**	**(95% CI**)	***p***	***padj***	N*	**(%**)	N*	**(%**)	**OR**	**(95% CI**)	***p***	***padj***
rs673768	GG	210	(76)	219	(72)	1		*0.422*	*0.973*	187	(70)	157	(72)	1		*0.949*	*1.000*
	AG	63	(23)	77	(25)	1.16	(0.79-1.71)			77	(29)	57	(26)	0.91	(0.61-1.38)		
	AA	5	(2)	7	(2)	1.21	(0.38-3.90)			3	(1)	4	(2)	1.78	(0.38-8.27)		
rs1804690	GG	217	(78)	264	(87)	1		***0.005***	***0.043***	211	(79)	186	(85)	1		***0.050***	*0.310*
	AG	60	(22)	40	(13)	0.55	(0.36-0.86)			53	(20)	31	(14)	0.66	(0.41-1.08)		
	AA	2	(1)	1	(0)	0.34	(0.03-3.86)			4	(1)	1	(0)	0.31	(0.03-2.82)		
rs3825061	GG	109	(39)	116	(38)	1		*0.744*	*1.000*	117	(44)	71	(33)	1		***0.001***	***0.010***
	AG	118	(42)	142	(47)	1.15	(0.80-1.64)			121	(45)	104	(48)	1.48	(0.99-2.21)		
	AA	52	(19)	47	(15)	0.86	(0.53-1.37)			29	(11)	43	(20)	2.53	(1.43-4.45)		
rs494048	GG	68	(29)	73	(28)	1		*0.784*	*1.000*	51	(22)	59	(32)	1		***0.004***	***0.034***
	AG	114	(48)	134	(52)	1.10	(0.73-1.67)			125	(54)	95	(52)	0.62	(0.39-1.00)		
	AA	55	(23)	53	(20)	0.91	(0.55-1.51)			55	(24)	29	(16)	0.42	(0.23-0.77)		
rs28990986	GG	239	(86)	255	(84)	1		*0.586*	*0.998*	230	(86)	187	(86)			*0.842*	*1.000*
	AG	36	(13)	48	(16)	1.29	(0.80-2.06)			35	(13)	30	(14)				
	AA	4	(1)	2	(1)	0.52	(0.09-2.88)			2	(1)	0	(0)				
rs640603	GG	106	(45)	109	(42)	1		*0.736*	*1.000*	79	(34)	99	(54)	1		***0.00004***	***0.001***
	AG	98	(41)	117	(45)	1.16	(0.79-1.69)			106	(46)	64	(35)	0.47	(0.30-0.72)		
	AA	33	(14)	34	(13)	1.01	(0.58-1.76)			46	(20)	20	(11)	0.34	(0.19-0.63)		
rs2509049	GG	83	(35)	87	(33)	1		*0.944*	*1.000*	58	(25)	80	(44)	1		***0.00003***	***0.001***
	AG	106	(45)	125	(48)	1.14	(0.76-1.70)			110	(48)	73	(40)	0.45	(0.29-0.72)		
	AA	48	(20)	49	(19)	0.98	(0.59-1.62)			63	(27)	30	(16)	0.33	(0.19-0.58)		
rs7759	AA	94	(40)	101	(39)	1		*0.970*	*1.000*	75	(32)	93	(51)	1		***0.00006***	***0.001***
	GA	106	(45)	122	(47)	1.08	(0.73-1.58)			104	(45)	67	(37)	0.49	(0.32-0.76)		
	GG	37	(16)	38	(15)	0.97	(0.57-1.65)			52	(23)	23	(13)	0.35	(0.19-0.63)		
rs8551	GG	94	(34)	102	(34)	1		*0.882*	*1.000*	76	(29)	91	(42)	1		***0.0005***	***0.006***
	AG	128	(46)	143	(47)	1.04	(0.72-1.51)			121	(45)	89	(41)	0.59	(0.39-0.89)		
	AA	57	(20)	59	(19)	0.95	(0.60-1.51)			69	(26)	37	(17)	0.43	(0.26-0.71)		
rs643788	AA	91	(33)	101	(33)	1		*0.964*	*1.000*	74	(28)	91	(42)	1		***0.0003***	***0.003***
	GA	132	(47)	142	(47)	0.98	(0.68-1.42)			124	(46)	89	(41)	0.56	(0.37-0.85)		
	GG	56	(20)	62	(20)	0.99	(0.63-1.57)			70	(26)	37	(17)	0.41	(0.25-0.68)		
rs604714	CC	109	(39)	119	(39)	1		*0.995*	*1.000*	94	(35)	104	(48)	1		***0.0008***	***0.008***
	AC	126	(45)	137	(45)	1.00	(0.70-1.43)			118	(44)	87	(40)	0.63	(0.42-0.95)		
	AA	44	(16)	48	(16)	1.00	(0.62-1.63)			56	(21)	27	(12)	0.42	(0.24-0.73)		
rs649870	AA	90	(32)	100	(33)	1		*0.918*	*1.000*	74	(28)	88	(40)	1		***0.0004***	***0.005***
	GA	133	(48)	144	(47)	0.99	(0.68-1.43)			121	(45)	92	(42)	0.61	(0.40-0.93)		
	GG	56	(20)	61	(20)	0.98	(0.61-1.55)			73	(27)	38	(17)	0.42	(0.25-0.69)		
rs571445	AA	91	(33)	94	(31)	1		*0.764*	*1.000*	71	(26)	88	(40)	1		***0.0002***	***0.003***
	GA	133	(48)	150	(49)	1.10	(0.76-1.59)			126	(47)	94	(43)	0.58	(0.38-0.89)		
	GG	55	(20)	60	(20)	1.06	(0.66-1.69)			71	(26)	36	(17)	0.39	(0.24-0.66)		

* The sum of the genotypes is in some cases lower than the total number of samples for a subtype, because some samples failed Illumina genotyping for some markers.

Abbreviations: Abbreviations: OR, odds ratio; CI, confidence interval

### Haplotype analysis

To determine if there is a specific *H2AFX* haplotype associated with NHL, linkage disequilibrium was examined between the 13 SNPs in all cases and controls in the BC population (Figure 1). Two haplotype blocks were predicted: Block 1, encompassing 2 SNPs in a 16 Kb region 3 Kb downstream of *H2AFX* and Block 2, encompassing the 7 SNPs in the 6 Kb region surrounding and directly upstream of *H2AFX* that show the most significant association with NHL. As neither of the SNPs in Block 1 was associated with NHL, this block was not analyzed further. For Block 2, haplotype associations with B-cell NHL as a whole and in females only were assessed (Table S4). In neither analysis was any one haplotype more significantly associated with NHL than the individual SNPs.

### Replication in independent study populations

**Figure 2:**
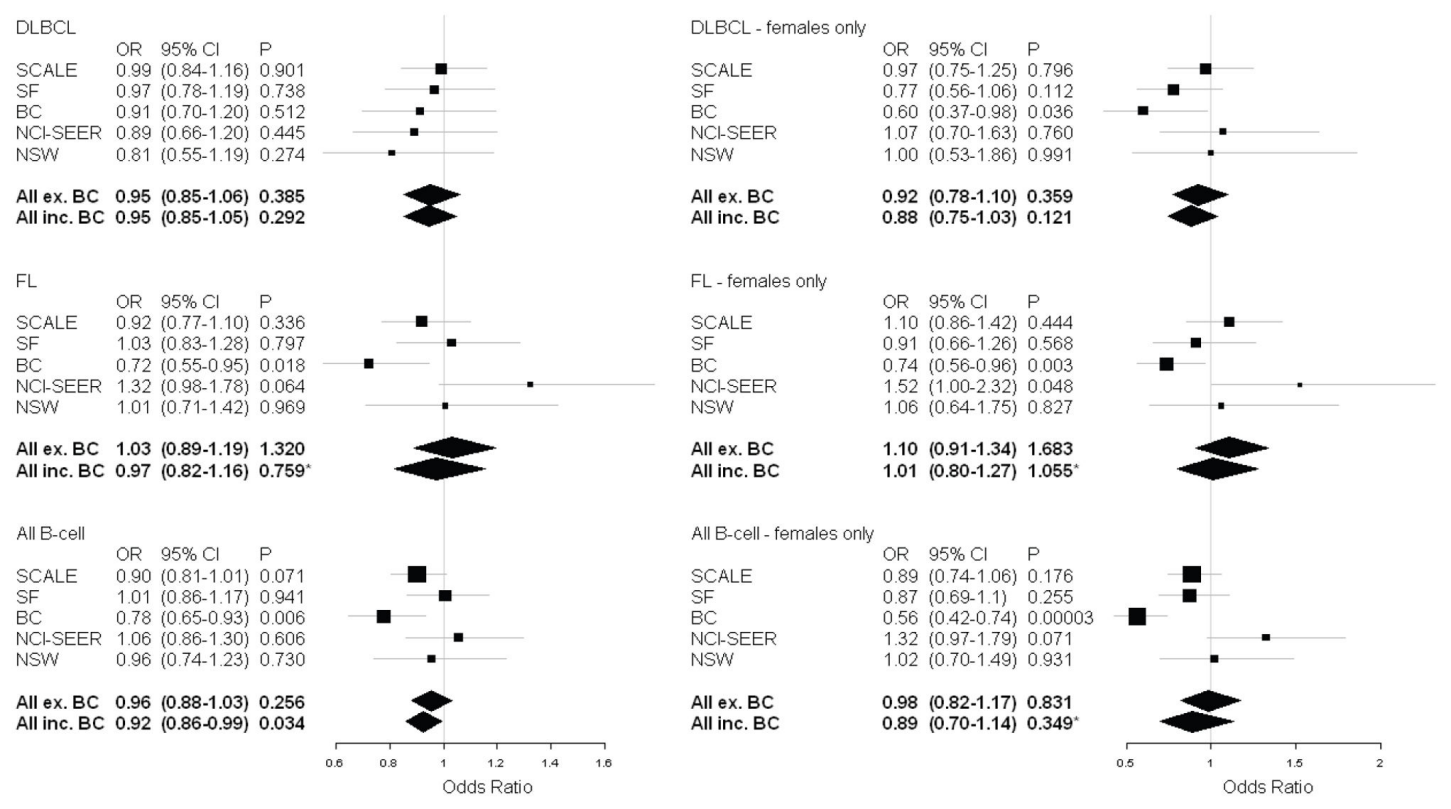
Forest plots for association of rs2509049 with DLBCL, FL and all B-cell lymphomas. Squares indicate the ORs, with the sizes proportional to the weight of the study in the meta-analysis. Summary ORs with and without the inclusion of the BC population are indicated in bold and designated with a diamond extending the width of the CI. All p values are from a fixed effects model except those indicated with an asterisk (*) which are from a random effects model. *Q* values for analyses including the BC dataset for DLBCL, FL and All B cell groups are *Q*=0.876, *Q*=0.053 and *Q*=0.161 for combined sexes, and *Q*=0.344, *Q*=0.045 and *Q*=0.002 for females only, respectively. Abbreviations: OR, odds ratio; 95% CI, 95% confidence interval; DLBCL, diffuse large B-cell lymphoma; FL, follicular lymphoma; all B-cell, all B-cell lymphomas; SCALE, Scandinavian Lymphoma Etiology; SF, San Francisco; BC, British Columbia; NCI-SEER, National Cancer Institute - Surveillance, Epidemiology and End Results; NSW, New South Wales. Figure created with rmeta version 2.16.

To replicate the sex- and subtype-specific association of *H2AFX* SNPs, rs2509049 allele frequencies were examined in 4 additional independent sample sets of NHL patients and controls from studies in the InterLymph Consortium. Details on the samples and genotyping protocols for these studies are summarized in Table S2. The rs2509049 association results for the DLBCL and FL subtypes and the all B cell group for males and females combined and for the female subset alone in the validation study populations are shown in Table S5 and summarized in Figure 2. The BC population was the only study to show a significant protective effect for the A allele; furthermore, the NCI-SEER population showed an association in the opposite direction that was statistically significant for the female FL subtype. Meta-analysis combining all 5 studies supported a significant but weak protective effect for the A allele only in the all B-cell group, however, this association was not significant with exclusion of the BC population.

## Discussion

Analysis of 13 SNPs within 100 Kb of *H2AFX* in the BC population supports previous reports [10,13] that variants in this region are associated with protection against B-cell NHL. In the BC population the association is confined to the FL and MCL subtypes and also appears to be sex-specific. Meta-analysis of a collective 3,882 NHL cases of B-cell origin from five populations showed a significant association with all B-cell lymphomas only in the combined male and female analysis, an effect that was not significant with exclusion of the BC sample set.

The BC population showed evidence that the association between *H2AFX* polymorphisms and B-cell lymphoma was sex-specific, present only in females and absent from the male subset. NHL has a higher incidence in males with an overall male to female incidence rate ratio of 1.6 for B-cell NHL [28] a phenomenon that may be due to a protective influence of female hormones on lymphomagenesis. Epidemiological studies reporting decreased NHL risk with increased parity [29,30], hormone contraceptive use [30,31] and hormone replacement therapy [32-35] provide support for this hypothesis. It is conceivable that female hormones directly or indirectly influence expression of *H2AFX* in an allele-specific manner. DNA repair capacity was noted to significantly decrease in cultured lymphocytes from females but not males older than age 48 [36]; however this finding was not replicated in a larger sample set [37] and no association with sex was seen with γH2AX response [37].

Sex-specificity was not evaluated in the previously reported associations between *H2AFX* and NHL [10,13], though the association between *H2AFX* variants and glioma in the Chinese Han population is reportedly stronger in male subjects [12]. Interestingly, the *H2AFX* association with glioma occurs in the opposite direction; the rs643788 A allele confers a protective effect for glioma [12], while our results suggest the G allele is protective for NHL. This phenomenon may be due to *H2AFX* promoter variants having opposing effects in different cell types, or differing roles for H2AX in development of these cancers.

It remains unclear whether there are subtype-specific associations between *H2AFX* and NHL. In the BC population, the association was significant only in the FL and MCL subtypes, though a trend toward reduced risk was also seen in DLBCL. Chromosomal translocations are found in 85-90% of FL [38] and nearly 100% of MCL tumors [39], but are less frequent in other NHL subtypes; they are present in 30-40% of DLBCL [40] and 10-50% of MZL [41]. The association of *H2AFX* genetic variants with translocation-prone lymphoma subtypes supports the hypothesis that H2AX is required for optimal resolution of double-stranded breaks introduced during B-cell development. Though the validation datasets only had sufficient numbers for a meta-analysis of DLBCL and FL subtypes, there was no evidence that the trend was stronger in the FL subtype. Furthermore, *H2AFX* polymorphisms were associated with protection against DLBCL in a Korean population [13] supporting the suggestion that the influence of *H2AFX* variants may extend to a variety of B-cell lymphoma subtypes.

Testing rs2509049, the SNP with the most significant effect in the all B-cell group, in four independent NHL patient collections did not replicate the observed association. This may indicate the observed association in the BC dataset was due to chance. However, the apparent sex-specificity of the *H2AFX*-NHL association may also provide an explanation for these differences. Differences in parity, use of hormonal contraceptives, postmenopausal hormone replacement therapy and exposure to estrogenic organochlorine pollutants between study regions could contribute to the differences observed. Fertility rates for the years 1980-85 are lower in Canada (1.63), Sweden (1.65) and Denmark (1.43) than in the USA (1.8) and Australia (1.91) [42]. Alternatively, although all replication populations were of European descent or white race, there may be undetected differences in genetic ancestry between studies that could explain the lack of consistency of association.

Though the effect was strongest in the 1.5 Kb immediately upstream of *H2AFX*, due to the high LD between SNPs in individuals of European ancestry, we were unable to determine which of the SNPs in this region (rs2509049, rs7759, rs8551, or rs643788), if any, is responsible. It is also possible that these SNPs are in LD with an undetected variant that is responsible for the association. The fact that haplotype analysis did not reveal an association more significant than that of individual SNPs, and that our previous resequencing of the *H2AFX* gene and upstream region in 95 NHL cases found no evidence for frequent rare mutations [10] make this explanation unlikely, unless the undetected SNP is either downstream or more than 1 Kb upstream of *H2AFX*. The lower LD between SNPs in this region in different ethnic populations may assist in determining which variant is responsible. For example, the Korean population has high LD (*r* ^2^=0.86) between rs643788 and rs8551 but lower LD between these SNPs and rs2509049 (*r* ^2^=0.78 and *r* ^2^=0.79, respectively); an association with DLBCL in this population was significant for rs8551 and rs643788, but not rs2509049 [13], suggesting that rs8551 and/or rs643788 may be relevant functional variants.

As the variants most strongly associated with NHL are located just upstream of the *H2AFX* gene, it is tempting to speculate that they influence gene expression by impacting transcription factor binding and altering promoter efficiency. An inspection of the DNA sequence at these sites revealed that the rs643788 G allele disrupts a consensus binding site for Yin-yang 1 (YY1) [43], a transcription factor capable of both activation and repression depending on cellular context [44]. Over-expression of YY1 is associated with tumor progression and poor outcome in NHL [45-47], consistent with a hypothesis that attenuated YY1 binding at *H2AFX* rs643788 is associated with reduction in cancer risk. However, other studies have made different predictions regarding the influence of these variants on binding site capacity: rs643788 is predicted to disrupt *CJUN* [11]; rs8551 is predicted to influence insulin activator factor [11] and CAP1 [13] binding; and the rs7759 G allele is predicted to disrupt a progesterone receptor binding site [11]. The latter prediction is particularly intriguing given the sex-specificity we report. Functional studies are required to determine which of these binding site predictions are supported by experimental evidence, and whether altered protein binding at these sites influences *H2AFX* gene expression.

rs1804690 was found to be associated with lymphoma risk in the all B-cell group. This association appears to be driven by a protective effect of the minor A allele in DLBCL and FL subtypes and is not sex-specific. As rs1804690 is more than 40 Kb downstream of *H2AFX* and not in LD with variants in the *H2AFX* region, it is unlikely (though not out of the question) that this association reflects an impact of rs1804690 on *H2AFX* expression or function. rs1804690 is a synonymous SNP located within exon 13 of the *HYOU1* gene. It may influence regulation of *HYOU1* or other genes in the region or be in LD with a variant that does so. *HYOU1* encodes Hypoxia Up-regulated 1, an oxygen regulated protein that may act as a molecular chaperone required in the cellular response to hypoxia. HYOU1 overexpression has been reported in breast [48] and colorectal cancer [49] tumors, and is associated with poor prognosis and metastasis into the lymphatic system [49]. Further research into the possible association of rs1804690 and B-cell NHL and a potential role for *HYOU1* in lymphomagenesis would be required to confirm this relationship.

## Conclusion

The combined results of 5 different NHL case-control studies suggest that overall DNA polymorphisms at *H2AFX* have a weak but significant association with NHL of B-cell origin; however this effect was largely driven by the BC sample set. The significant result in the BC dataset may be a spurious finding or suggest this variant has an impact unique to the lifestyle factors or genetic background in this population. Given the biological importance of H2AFX in cancer, further research is warranted to understand the effects of genetic variation at this locus, both functionally and in human populations. 

## Supporting Information

Table S1 **SNPs selected for genotyping in the BC population.**
(XLSX)

Table S2 **Characteristics of study populations included in meta-analysis.**
(XLSX)

Table S3 **Subtype-specific sex-SNP interaction analysis in the BC population.**
(XLSX)

Table S4 **Sex-specific association results for haplotype block 2 in the BC population.**
(XLSX)

Table S5 **Subtype-specific association results for rs2509049 in replication populations.**
(XLSX)
